# Effect of Dezocine on Hemodynamic Indexes of Postoperative Patients With Traumatic Brain Injury (TBI)---A Pilot Study

**DOI:** 10.3389/fphar.2022.665107

**Published:** 2022-03-31

**Authors:** Xuejian Wang, Yang Chen, Zhifeng Wang, Yi Zhang, Zhiming Cui, Chen Sun

**Affiliations:** Department of Neurosurgery, Affiliated Hospital 2 of Nantong University, Nantong University, Nantong, China

**Keywords:** dezocine, hemodynamics, complications, traumatic brain injury (TBI), postoperative

## Abstract

**Background:** Due to pain and other stimuli, patients with traumatic brain injury (TBI) after surgery show excited Sympathetic Nervous system, increased intracranial pressure, brain tissue swelling, intracranial hemorrhage, or reduced cerebral perfusion pressure, seriously threatening the life and prognosis of patients. The effect of dezocine on postoperative analgesia after TBI remains largely undetermined.

**Objective:** In the present study, we aimed to investigate the efficacy and safety of dezocine in postoperative sedative and analgesic therapy for a craniocerebral injury.

**Methods:** The patients were randomly divided into two groups (n = 40) as follows: dezocine group (Group A) and control group (Group B). Electrocardiography (ECG), heart rate (HR), blood pressure, and oxygen saturation (SpO_2_) were routinely monitored after postoperative return to the ward. Both groups were initially injected with 5 mg·kg^−1^·h^−1^ propofol to maintain sedation, and the dose was adjusted according to the patient’s condition. Vital signs of patients were recorded at T1 (the base value when arriving at the ward), T2 (before the sedative agent was used) and T3 (use of dezocine or 0.9% saline solution for 8 h), T4 (use for 1 day), T5 (use for 3 days), T6 (termination of dezocine or 0.9% saline solution for 1 day), and T7 (termination for 3 days), and mean arterial pressure (MAP) and HR values were also recorded. The total amount of propofol, total fluid inflow, blood loss, and urine output were recorded within 24 h. The number of coughs of each patient was recorded within 1 day after entry, and the incidence of adverse events, such as insufficient oxygenation (SaO_2_ reduced by about 5% from the base value), hypotension, bradycardia, laryngospasm, bronchospasm, and so on, was assessed.

**Results:** Compared with the control group (group B), the hemodynamics of the dezocine group (group A) was more stable, there were significant differences in MAP and HR (*p* < 0.05), and the stress response was milder. The total amount of propofol, total fluid inflow, blood loss, and urine volume of the dezocine group were significantly improved compared with the control group (*p* < 0.05). Moreover, the incidence of adverse events, such as cough, in the dezocine group was significantly reduced compared with the control group (*p* < 0.05).

**Conclusions:** Dezocine, as a drug with a strong analgesic effect and obvious sedative effect, was suitable for craniocervical surgery, and it could significantly improve the stability of airway and hemodynamics in TBI patients during anesthesia recovery.

## Introduction

In the peri-anesthetic period, patients with traumatic brain injury (TBI) will be excited by various stimuli, resulting in increased intracranial pressure, brain tissue swelling, intracranial hemorrhage, or reduced cerebral perfusion pressure, seriously threatening the life and prognosis of patients (Lafrenaye et al., 2012; Wiener et al., 2019). Therefore, it is particularly necessary to reduce adverse stimulation and sympathetic overexcitement, decrease metabolism and oxygen consumption, lessen injurious stimulation, and lower excessive hemodynamic changes caused by stress factors in TBI patients. (Fragen and Caldwell, 1978; Ramirez-Ruiz et al., 1995; Han et al., 2016; Wiener et al., 2019). As an opioid receptor mixed agonist-antagonist with a strong analgesic effect, dezocine has been widely used in various pain treatments (Fragen and Caldwell, 1978; Sun et al., 2012; Liu et al., 2014; Li et al., 2019). Pain after TBI in neurosurgery is a common phenomenon. There have been few reports and related studies on dezocine in neurosurgery (Lafrenaye et al., 2012; Han et al., 2016; Wiener et al., 2019). However, the effect of dezocine on TBI patients has not been well studied. From this study, we observe the effect of dezocine on hemodynamic indexes of TBI patients during anesthesia recovery.

## Materials and Methods

Thi s study was approved by the Ethics Committee of our hospital, and written informed consent was obtained from all patient guardians.

### General Information

Inclusion criteria were set as follows: patients with Glasgow Coma Scale (GCS) of 5–12 points, age of 18–60 years, and body mass of 50–80 kg. The types of craniocerebral injury included brain contusion, skull fracture, intracranial hematoma, subdural hematoma, and epidural hematoma. There was no shock before or during the operation. There was no cerebral hernia. Craniocerebral surgery was required. Patient guardians signed the consent form.

From February 2018 to January 2020, 80 patients with moderate and severe TBI who received emergency treatment from the neurosurgery department in our hospital were selected, and the injury time ranged from 6 to 24 h. There were 44 males and 36 females, and their average age was 37.23 ± 9.56 years, ranging from 18 to 56 years. The above-mentioned patients were randomly and evenly divided into the dezocine group (group A) and control group (group B) using the random number method. There was no statistically significant difference in general data between the two groups (Table 1).

**TABLE 1:** Comparison of general condition indexes and operation time between the two groups (n = 40).

Group		Group A	Group B
Age		37.89 ± 9.56	38.22 ± 9.21
Body mass (kg)		64.17 ± 8.24	63.64 ± 8.72
Male/female		21/19	17/23
ASA (Level I/II)		12/28	14/26
GCS score (case)	5˜8	17	16
	9˜12	23	24
Time of operation (min)		186.24 ± 61.47	172.13 ± 57.24

Notes: There was no statistically significant difference in general data between the two groups. dezocine group (Group A); control group (Group B).

### Anesthesia Methods

Electrocardiography (ECG), heart rate (HR), blood pressure, and oxygen saturation (SpO_2_) were routinely monitored after the patient returned to the ward. Both groups were initially injected with 5 mg·kg^−1^·h^−1^ propofol to maintain the sedation, and the dose was adjusted according to the patient’s condition. Meanwhile, patients in group A received 100 ml 0.9% sodium chloride containing 5 mg dezocine every 12 h. Patients in group B received 100 ml 0.9% sodium chloride every 12 h.

### Observation Indicators

Vital signs of patients were recorded at T1 (the base value when arriving at the ward), T2 (before the sedative agent was used) and T3 (use of dezocine or 0.9% saline solution for 8 h), T4 (use for 1 day), T5 (use for 3 days), T6 (termination of dezocine or 0.9% saline solution for 1 day), and T7 (termination for 3 days), and the mean arterial pressure (MAP) and HR values were also recorded. The total amount of propofol, total fluid inflow, blood loss, and urine output were recorded for 24 h. The number of coughs for each patient was recorded within 1 day after entry, and the incidence of adverse events, such as insufficient oxygenation (SaO_2_ reduced by about 5% from the base value), hypotension, bradycardia, laryngospasm, and bronchospasm, were also recorded.

### Statistical Analysis

SPSS 20.0 statistical software was used for analysis. Measurement data were expressed as (‾X ± S), and a Student’s t-test was used for pairwise comparison between groups. Besides, the chi-square test was used for comparison of count data. *p* < 0.05 was considered statistically significant.

## Results

### Changes of Hemodynamic Index

**FIGURE 1:**
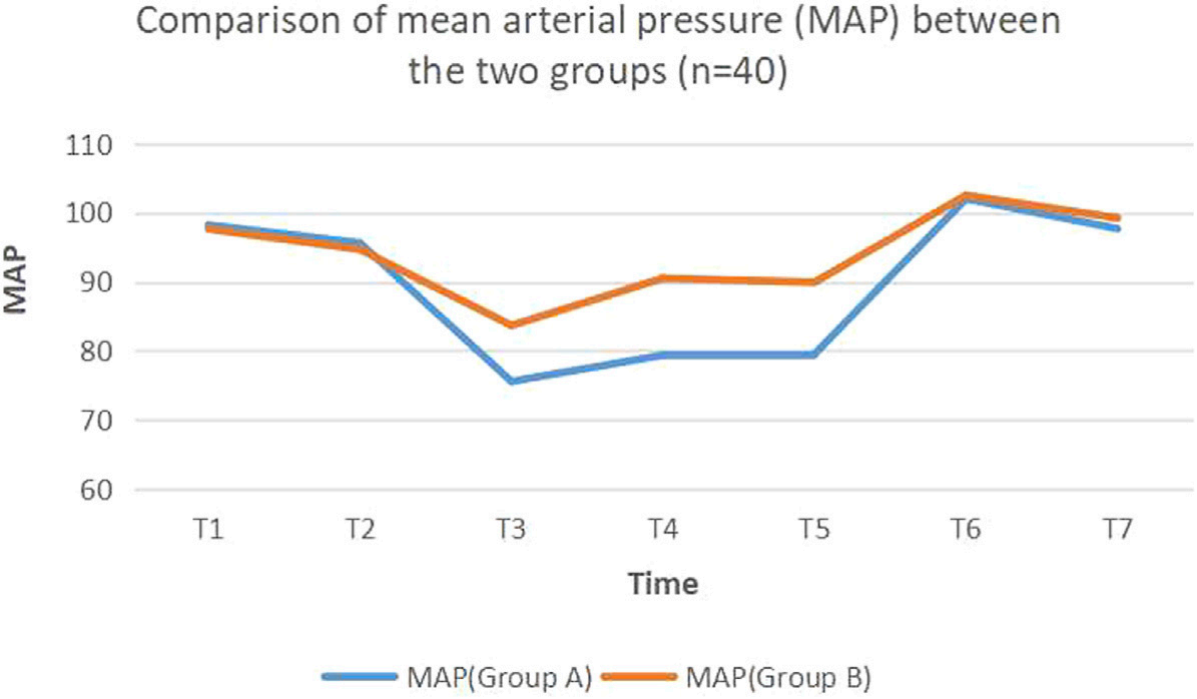
Comparison of mean arterial pressure (MAP) between.

**FIGURE 2:**
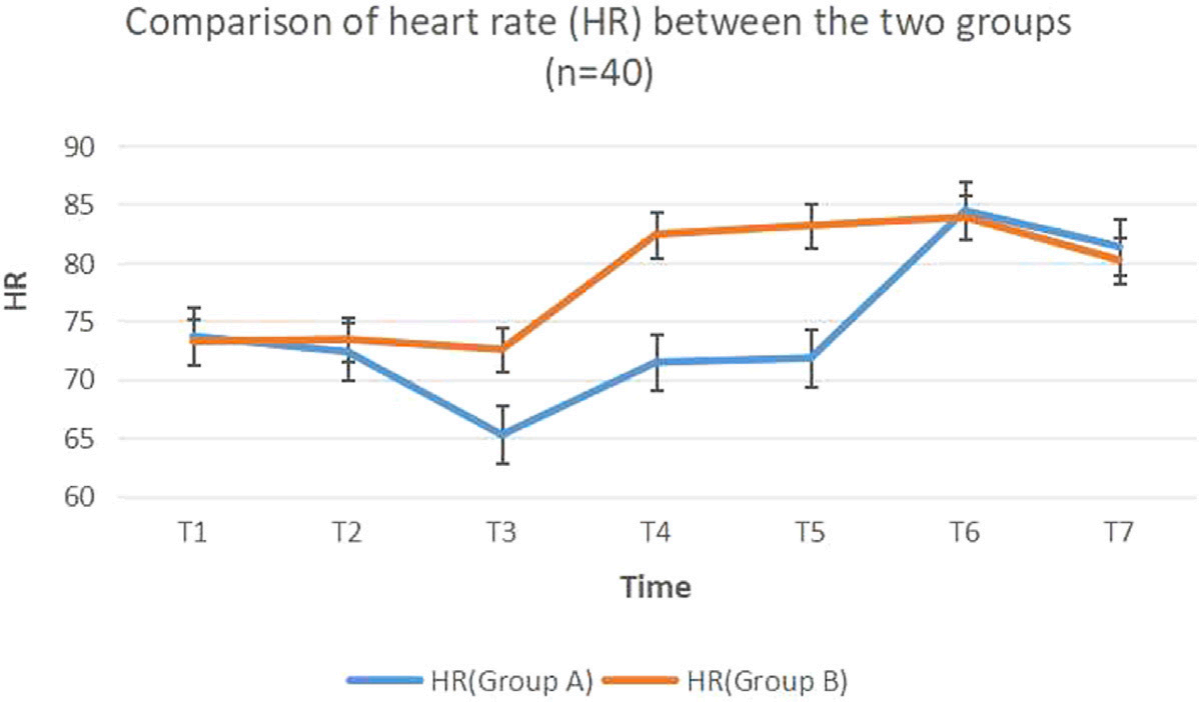
Comparison of heart rate (HR) between the two st.

After two groups of patients returned to the ward, the indexes of T1, T2, T6, and T7 had no difference, while the MAP of the dezocine group at other time points (T3, T4, and T5) was lower compared with the control group (t = 4.11, 5.42, 5.31, *p* < 0.05) (Table 2; Figure 1, Figure 2).

**TABLE 2:** Comparison of vital signs between the two groups (n = 40).

Type	Group	T1	T2	T3	T4	T5	T6	T7
MAP (mmHg)	Group A	98.23 ± 11.35	95.63 ± 9.82	75.54 ± 8.43	79.36 ± 8.72	79.52 ± 8.42	102.04 ± 9.24	97.69 ± 8.94
	Group B	97.65 ± 12.87	94.68 ± 10.13	83.67 ± 9.21	90.51 ± 9.67	89.94 ± 9.13	102.54 ± 11.53	99.24 ± 9.67
HR (Times/min)	Group A	73.68 ± 8.64	72.37 ± 9.12	65.26 ± 8.37	71.47 ± 9.13	71.82 ± 9.34	84.43 ± 9.62	81.37 ± 9.67
	Group B	73.26 ± 9.13	73.42 ± 8.94	72.58 ± 8.34	82.41 ± 9.45	83.17 ± 9.81	83.87 ± 10.03	80.23 ± 9.92

Notes: the indexes of T1, T2, T6, and T7 had no difference, while the MAP, of the dezocine group at other time points (T3, T4, and T5) was lower compared with the control group (t = 4.11, 5.42, 5.31, *p* < 0.05). dezocine group (Group A); control group (Group B); T1 (the base value when arriving at the ward), T2 (before the sedative agent was used) and T3 (use of dezocine or 0.9% saline solution for 8 h), T4 (use for 1 day), T5 (use for 3 days), T6 (termination of dezocine or 0.9% saline solution for 1 day), and T7 (termination for 3 days).

### Comparison of the Amount of Propofol Used and the Amount of Liquid in and out Between the Two Groups

The amount of propofol used in the dezocine group was significantly lower compared with the control group (t = 12.58, both *p* < 0.05). There was no statistically significant difference in blood loss, urine volume, and fluid inflow between the two groups (*p* > 0.05) (Table 3).

**TABLE 3:** Comparison of propofol usage and fluid inflow and outflow between the two groups (N = 40, X ± s).

Group	Usage of propofol	Amount of bleeding	Urine volume	Liquid intake
	mg・kg^−1^・h^−1^	(ml/h)	(ml/h)	(ml/h)
Group A	4.27 ± 0.58	3.28 ± 0.23	26.22 ± 0.58	60.23 ± 0.82
Group B	6.35 ± 0.87	3.24 ± 0.19	25.17 ± 0.62	60.17 ± 0.76

Notes: The amount of propofol used in the dezocine group was significantly lower compared with the control group (t = 12.58, both *p* < 0.05). There was no statistically significant difference in blood loss, urine volume, and fluid inflow between the two groups (*p* > 0.05). dezocine group (Group A); control group (Group B).

### Comparison of Respiratory Events and Other Results Between the Two Groups

One and eight cases of cough were observed in the dezocine group and control group within 1 day after the operation (F = 4.02, *p* < 0.05), respectively. Moreover, one and three cases in the dezocine group and control group had insufficient oxygenation, respectively, which was improved after oxygen flow was increased or the mask was changed to take oxygen. There were two cases and one case of postoperative hypotension in the dezocine group and control group, respectively, both of which were improved after intravenous fluids or vasopressors were administered. Bradycardia occurred in one case in each group, which was improved by dopamine treatment. There was no laryngospasm or bronchospasm in both groups (Table 4).

**TABLE 4:** Comparison of postoperative respiratory events and other results between the two groups (n = 80,%).

Group	Num	Cough	Insufficient oxygenation	Hypotension	Bradycardia	Laryngospasm	Bronchospasm
Group A	40	1 (2.5)	1 (2.5)	2 (5)	1 (2.5)	0 (0.00)	0 (0.00)
Group B	40	8 (20)	3 (7.5)	1 (2.5)	1 (2.5)	0 (0.00)	0 (0.00)

Notes: One and eight cases of cough were observed in the dezocine group and control group within 1 day after the operation (t = 4.02, *p* < 0.05), respectively. dezocine group (Group A); control group (Group B).

## Discussion

TBI patients should reduce the metabolic level and oxygen consumption as much as possible after surgery to reduce further damage caused by strong stressors. (Sharma and Vavilala, 2012; Zhou et al., 2015). Sedation and analgesia are the main measures to improve postoperative stress response. (Urwin and Menon, 2004; Woldegerima et al., 2016). Currently known sodium thiopental, propofol, midazolam, etomidate, and so on can not prevent and reduce the stress response. However, traditional opioid analgesics, such as morphine and fentanyl, have strong analgesic effects, which can reduce the postoperative stress response. However, they also have adverse reactions, such as respiratory depression, nausea, and vomiting, leading to an increased risk of postoperative complications in TBI patients. If a tranquilizer drug is used, it may put the patient back into a deep sleep, accompanied by residual anesthesia, resulting in an elevated risk of respiratory depression or aspiration.

Dezocine is a new type of compound agonist-antagonist of opioid receptors. As a phenylmorphine derivative, it mainly excites the receptors in the brain, brain stem, and spinal cord, leading to analgesic and mild sedative effects. Meanwhile, it exerts partial excitatory and antagonistic effects on the receptor. Therefore, dezocine does not result in typical receptor dependence but has a strong analgesic effect and obvious sedative effect (Sun et al., 2012). Moreover, it does not induce fidgeting and anxiety, showing fewer clinical adverse reactions. (An et al., 2017). A great deal of evidence supports that κ opioid receptor (KOR) plays a significant role in the development of µ opioid receptor (MOR)–mediated opioid dependence, tolerance, and withdrawal. (Spanagel et al., 1994; Bolanos et al., 1996; Wang et al., 2010). Dezocine in combination with opioid agonists and antagonists exhibits a stronger analgesic effect compared with morphine and codeine alone. One study (de Nadal et al., 2000) has reported that a bolus injection of morphine is associated with a significant increase in ICP and significant decreases in CPP and MAP.

Dezocine has a strong analgesic effect due to its unique pharmacological action, and its side effect is very slight. Therefore, it is an ideal analgesic drug for intravenous use. We showed that compared with the control group, dezocine could reduce the postoperative MAP and HR fluctuation (*p* < 0.05), effectively reduce the effect of stress factors, and help maintain the stability of the brain environment.

In traditional surgeries, propofol sedation, inhalation anesthesia, or opioid analgesics, is often used alone, which may result in overdose, increase cerebral blood flow, and even affect the recovery of postoperative respiratory function. (Chen et al., 1993). Some experts (Lauer et al., 1997) have reported a significant difference between opioids, showing that fentanyl infusion is associated with significantly lower ICP and CPP compared with an infusion of either morphine or sufentanil. In this study, the use of continuous propofol in combination with the adjuvant infusion of dezocine, as well as the use of analgesics, reduced the dose of propofol and avoided the occurrence of complications, such as respiratory depression, bradycardia, and hypotension (Wu et al., 2019). We further compared the amount of propofol used and the amount of liquid in and out between the two groups. We found that the amount of propofol used in the dezocine group was significantly less compared with the control group.

From the perspective of complications, the use of sedative agents and opioids has been extensively reported in the literature. (Pfeiffer et al., 1986; Suzuki et al., 1992; Zhang et al., 2005; Carlezon et al., 2006; Bruchas et al., 2007). Choking and hypoxemia are the most common side effects of anesthesia postoperative TBI, as well as hypertension, tachycardia, elevated intracranial pressure, increased myocardial oxygen consumption, bronchospasm, and postoperative bleeding. In the present study, we found that the application of dezocine after surgery significantly reduced the incidence of cough reflex, and the adverse reactions in the dezocine group were significantly improved compared with the control group. These findings indicated that dezocine could make patients tolerate intubation, and significantly reduce postoperative cough, leading to remarkably attenuated adverse reactions. This might be closely related to the sedative and analgesic effects of dezocine itself.

## Conclusion

Collectively, dezocine, as a drug with a strong analgesic effect and obvious sedative effect, was suitable to be used after craniocerebral surgery to reduce the incidence of adverse reactions, such as choking and agitation. Dezocine could significantly improve the stability of the airway and hemodynamics of TBI patients during postoperative recovery from anesthesia, thus improving the comfort level and vital signs of patients.

## Data Availability

The original contributions presented in the study are included in the article/Supplementary Material, further inquiries can be directed to the corresponding author.

## References

[B1] An L. J. Zhang Y. Su Z. Zhang X. L. Liu H. L. Zhang Z. J. (2017). A Single Dose of Dezocine Suppresses Emergence Agitation in Preschool Children Anesthetized with Sevoflurane-Remifentanil. BMC Anesthesiol 17 (1), 154. 10.1186/s12871-017-0446-8 29166854 PMC5700567

[B2] Bolanos C. A. Garmsen G. M. Clair M. A. McDougall S. A. (1996). Effects of the Kappa-Opioid Receptor Agonist U-50,488 on Morphine-Induced Place Preference Conditioning in the Developing Rat. Eur. J. Pharmacol. 317 (1), 1–8. 10.1016/s0014-2999(96)00698-x 8982712

[B3] Bruchas M. R. Land B. B. Aita M. Xu M. Barot S. K. Li S. (2007). Stress-induced P38 Mitogen-Activated Protein Kinase Activation Mediates Kappa-opioid-dependent Dysphoria. J. Neurosci. 27 (43), 11614–11623. 10.1523/JNEUROSCI.3769-07.2007 17959804 PMC2481272

[B4] Carlezon W. A. Jr Béguin C. DiNieri J. A. Baumann M. H. Richards M. R. Todtenkopf M. S. (2006). Depressive-like Effects of the Kappa-Opioid Receptor Agonist Salvinorin A on Behavior and Neurochemistry in Rats. J. Pharmacol. Exp. Ther. 316 (1), 440–447. 10.1124/jpet.105.092304 16223871

[B5] Chen J. C. Smith E. R. Cahill M. Cohen R. Fishman J. B. (1993). The Opioid Receptor Binding of Dezocine, Morphine, Fentanyl, Butorphanol and Nalbuphine. Life Sci. 52 (4), 389–396. 10.1016/0024-3205(93)90152-s 8093631

[B6] de Nadal M. Munar F. Poca M. A. Sahuquillo J. Garnacho A. Rosselló J. (2000). Cerebral Hemodynamic Effects of Morphine and Fentanyl in Patients with Severe Head Injury: Absence of Correlation to Cerebral Autoregulation. Anesthesiology 92 (1), 11–19. 10.1097/00000542-200001000-00008 10638893

[B7] Fragen R. J. Caldwell N. (1978). Comparison of Dezocine (WY 16, 225) and Meperidine as Postoperative Analgesics. Anesth. Analg 57 (5), 563–566. 10.1213/00000539-197857050-00010 30343

[B8] Han J. Yang S. Zhang C. Zhao M. Li A. (2016). Impact of Intracranial Pressure Monitoring on Prognosis of Patients with Severe Traumatic Brain Injury: A PRISMA Systematic Review and Meta-Analysis. Medicine (Baltimore) 95 (7), e2827. 10.1097/MD.0000000000002827 26886639 PMC4998639

[B9] Lafrenaye A. D. McGinn M. J. Povlishock J. T. (2012). Increased Intracranial Pressure after Diffuse Traumatic Brain Injury Exacerbates Neuronal Somatic Membrane Poration but Not Axonal Injury: Evidence for Primary Intracranial Pressure-Induced Neuronal Perturbation. J. Cereb. Blood Flow Metab. 32 (10), 1919–1932. 10.1038/jcbfm.2012 22781336 PMC3463883

[B10] Lauer K. K. Connolly L. A. Schmeling W. T. (1997). Opioid Sedation Does Not Alter Intracranial Pressure in Head Injured Patients. Can. J. Anaesth. 44 (9), 929–933. 10.1007/BF03011963 9305555

[B11] Li X. T. Ma C. Q. Qi S. H. Zhang L. M. (2019). Combination of Propofol and Dezocine to Improve Safety and Efficacy of Anesthesia for Gastroscopy and Colonoscopy in Adults: A Randomized, Double-Blind, Controlled Trial. World J. Clin. Cases 7 (20), 3237–3246. 10.12998/wjcc.v7.i20.3237 31667174 PMC6819283

[B12] Liu R. Huang X. P. Yeliseev A. Xi J. Roth B. L. (2014). Novel Molecular Targets of Dezocine and Their Clinical Implications. Anesthesiology 120 (3), 714–723. 10.1097/ALN.0000000000000076 24263237 PMC3944410

[B13] Pfeiffer A. Brantl V. Herz A. Emrich H. M. (1986). Psychotomimesis Mediated by Kappa Opiate Receptors. Science 233 (4765), 774–776. 10.1126/science.3016896 3016896

[B14] Ramirez-Ruiz M. Smith I. White P. F. (1995). Use of Analgesics during Propofol Sedation: a Comparison of Ketorolac, Dezocine, and Fentanyl. J. Clin. Anesth. 7 (6), 481–485. 10.1016/0952-8180(95)00058-p 8534465

[B15] Sharma D. Vavilala M. S. (2012). Perioperative Management of Adult Traumatic Brain Injury. Anesthesiol Clin. 30 (2), 3335–3346. 10.1016/j.anclin.2012.04.003 PMC3424485 22901613

[B16] Spanagel R. Almeida O. F. Bartl C. Shippenberg T. S. (1994). Endogenous Kappa-Opioid Systems in Opiate Withdrawal: Role in Aversion and Accompanying Changes in Mesolimbic Dopamine Release. Psychopharmacology (Berl) 115 (1-2), 121–127. 10.1007/BF02244761 7862883

[B17] Sun Q. Zhou W. Wu B. Ji M. H. Peng Y. G. (2012). Dezocine: a Novel Drug to Prevent Fentanyl-Induced Cough during General Anesthesia Induction? J. Anesth. 26 (3), 470. 10.1007/s00540-011-1318-x 22228499

[B18] Suzuki T. Shiozaki Y. Masukawa Y. Misawa M. Nagase H. (1992). The Role of Mu- and Kappa-Opioid Receptors in Cocaine-Induced Conditioned Place Preference. Jpn. J. Pharmacol. 58 (4), 435–442. 10.1254/jjp.58.435 1328733

[B19] Urwin S. C. Menon D. K. (2004). Comparative Tolerability of Sedative Agents in Head-Injured Adults. Drug Saf. 27 (2), 107–133. 10.2165/00002018-200427020-00003 14717622

[B20] Wang Y. H. Sun J. F. Tao Y. M. Chi Z. Q. Liu J. G. (2010). The Role of Kappa-Opioid Receptor Activation in Mediating Antinociception and Addiction. Acta Pharmacol. Sin 31 (9), 1065–1070. 10.1038/aps.2010.138 20729876 PMC4002313

[B21] Wiener J. McIntyre A. Janzen S. Mirkowski M. MacKenzie H. M. Teasell R. (2019). Opioids and Cerebral Physiology in the Acute Management of Traumatic Brain Injury: a Systematic Review. Brain Inj. 33 (5), 559–566. 10.1080/02699052.2019.1574328 30696281

[B22] Woldegerima N. Rosenblatt K. Mintz C. D. (2016). Neurotoxic Properties of Propofol Sedation Following Traumatic Brain Injury. Crit. Care Med. 44 (2), 455–456. 10.1097/CCM.0000000000001322 26771796 PMC6486399

[B23] Wu F. X. Babazada H. Gao H. Huang X. P. Xi C. H. Chen C. H. (2019). Dezocine Alleviates Morphine-Induced Dependence in Rats. Anesth. Analg 128 (6), 1328–1335. 10.1213/ANE.0000000000003365 31094808 PMC6173660

[B24] Zhang Y. Butelman E. R. Schlussman S. D. Ho A. Kreek M. J. (2005). Effects of the Plant-Derived Hallucinogen Salvinorin A on Basal Dopamine Levels in the Caudate Putamen and in a Conditioned Place Aversion Assay in Mice: Agonist Actions at Kappa Opioid Receptors. Psychopharmacology (Berl) 179 (3), 551–558. 10.1007/s00213-004-2087-0 15682306

[B25] Zhou X. Zhang C. Wang M. Yu L. Yan M. (2015). Dezocine for Preventing Postoperative Pain: A Meta-Analysis of Randomized Controlled Trials. PLoS One 10 (8), e0136091. 10.1371/journal.pone.0136091 26287536 PMC4545891

